# Wet ball milling of niobium by using ethanol, determination of the crystallite size and microstructures

**DOI:** 10.1038/s41598-021-01916-w

**Published:** 2021-11-17

**Authors:** Azunna Agwo Eze, Emmanuel Rotimi Sadiku, Williams Kehinde Kupolati, Jacques Snyman, Julius Musyoka Ndambuki, Tamba Jamiru, Mondiu Olayinka Durowoju, Idowu David Ibrahim, Mxolisi Brendon Shongwe, Dawood A. Desai

**Affiliations:** 1 Institute for Nano-Engineering Research (INER), Department of Chemical, Metallurgical and Materials Engineering, Tshwane University of Technology, Pretoria, South Africa; 2 Department of Civil Engineering, Tshwane University of Technology, Pretoria, South Africa; 3 Department of Mechanical Engineering, Mechatronics and Industrial Design, Tshwane University of Technology, Pretoria, South Africa; 4 Department of Mechanical Engineering, Ladoke Akintola University of Technology (LAUTEC), Ogbomoso, Oyo State Nigeria

**Keywords:** Materials science, Nanoscience and technology

## Abstract

This study investigates the effect of using ethanol as the process control agent during the wet ball milling of niobium (Nb). Dried nanocrystal Nb powders, of high purity, with particle sizes, ranging from 8.5 to 14.3 nm, were synthesized by ball milling. Commercial Nb powder of particle sizes of − 44 µm was employed by using the planetary ball mill equipped with stainless still vials with still balls in ethanol. A ball-to-powder mass ratio of 10:1 was used at a rotation speed of 400 rpm, an interval of 15 min with an interval break of 5 s, and a milling time of 10 h. The powder was dried in vacutec at a temperature of 100 °C, using a speed of 15 rpm in the vacuum of 250 mbar at a time of approximately 653 min. The crystal phase of the dried powders was analyzed using X-ray diffraction (XRD) with CuK_ɑ_ radiation, and by modification of the Scherrer equation, a single crystallite size of 11.85 nm was obtained. The morphology of the particles was observed using scanning electron microscopy (SEM) with energy-dispersive X-ray spectroscopy (EDS). The XRD results show that the pure crystal sizes in nanometre (nm), which decreases as the 2θ and the full width at half maximum (FWHM) increases.

## Introduction

Niobium is a body-centered cubic (bcc) crystallite structure, an essential micro-alloying metallic element for the pipeline steels, steel for structural applications, excellent alloys for aircraft turbine engines, car body steels with high strength, and it is in the utmost demand in the mechanized countries, which is now accounting to a sale capacity of more than 85,000 tonnes every year^1–4^. Niobium has exceptional ductility, a high melting point of 2740 K, oxidation and impact resistance, a density of 8.55 g/cm^3^, high dielectric dissipation, a small neutron-absorbing cross-sectional area, and a high transition temperature of 9.3 K (i.e. − 264 °C, or − 443 °F), among metals. Therefore, the metal is used widely, in nuclear fusion, nuclear industry, space development, high power transmission, and superconductor^4^.

The decrease in the particle size and the reduction in agglomeration increase the suspension stability of the alloyed materials^5^. Powder particle size has to be fine enough to gain a smooth surface finish and satisfactory optimal precision^6,7^. The reduction in particle size of metallic flakes or powders can be achieved by either dry milling or wet milling techniques. The better amongst the two techniques remain insignificant in small-scale milling, but there exists, the main practical problem when huge scale milling in metallurgical manufacturing, is required. In the wet milling technique, process control agents (lubricant or surfactant) such as: ethanol, stearic acid, methanol, hexane etc., are added to the powder mixture during milling to reduce the effect of cold welding^8^. The ethanol will function as surface-active agents, and will be absorbed on the surface of the powder particles, and minimize cold welding between niobium powder particles and thus slow down agglomeration. The ethanol would interfere with cold welding of the milling particles and therefore, lowered the surface tension of the powdered material. The wet milling process is the main technique used for commercial production^9^. In ancient times, solvents, e.g., ethanol has been used during the milling process to make metal flakes^10^. The solvent is much to offer wet milling of metallic powders to form chips. It has been made reported that the use of ethanol and other carbon- and oxygen-containing solvents for either wet-milling or wet-grinding of metal flakes to form powders can create several problems^10^. The oxygen present in the wet milling solvents can be released from the solvent due to the ease with which carbon to oxygen bonds are broken down. When oxygen comes in the system from the broken solvent molecules, the oxygen can react or be present with the metal chips being formed or with the stainless steel milling intermediate and cause impurities in the resulting chips^10^. It is assumed that rupture of carbon–oxygen bonds in the wet milling solvents, can leads to a high stage of carbon and iron contaminations in the chips produced and causes a consequential corrosive environment^10^. The issue of carbon or oxygen contamination depends mainly on further production methods (e.g., consolidation of the powdered particles) and the area of application of the final product. However, the annealing of the powders before the spark plasma sintering (SPS) powder consolidation techniques, can remove/reduce the oxygen contamination^11^ and if carbon is the major impurity in Nb, as in the case of this study, the powders produced will be mostly a transition metal carbides of niobium carbide (NbC) powders. NbC is an important material and it is often, added as a hard phase in composite materials^12^. Transition carbide (NbC) possesses a specific combination of thermal, mechanical and electrical conductivity properties, such as: high melting temperature, high hardness, good high-temperature strength and good electrical conductivity^13^. These materials are also used as high-temperature structural materials in the form of hard constituents in metal matrix composites^13–18^. NbC can find uses as structural materials that are resistant to high temperatures and corrosive atmospheres, like abrasives, supper conductors and high-performance permanent magnets^19^. In summary, the usefulness of transition carbide cannot be overemphasized.

XRD is a convenient method for determining the mean size of nanocrystallites in nanocrystallite bulk materials^20^. Paul Scherrer, one of the 1st scientist to work on X-ray diffraction, had his research results in a published paper that incorporated what turned out to be known as the Scherrer equation as shown in Eq. 1, in 1918^21^, which can be employed in the determination of the crystal size.

1 $$L = \frac{K\lambda }{{\beta \cdot \cos \theta }}$$

where *L*, represents the average crystallite size, *K* is 0.9 (the Scherrer constant or shape factor), and it is related to crystal shape. ʎ = 0.15405 nm (the CuK_α1_ wavelength), β is the full width at half maximum (FWHM), whose value on the 2θ axis of the diffraction profile, must be in radian. θ, the Bragg angle is the value of half the angle between the transmitted and reflected beams, 2θ in degrees or radians; since the cosine values of a number in radians correspond to the same value in degree.

In the work of Monshi et al.^20^, the Scherrer equation was modified to provide a new approach to the use of the Scherrer equation, so that the least square technique can be applied to minimize the source of error. Their work established the modified Scherrer equation, which was a plot of ℓnβ as a function of ℓn(1/Cosθ) and an intercept is obtained of a least square regression, $\ln = \frac{K\lambda }{L}$, from which a single value of crystal size, *L*, was obtained through all of the available peaks^20^.

On the other hand, the current study examines the use of ethanol as a process control agent, used to mill pure niobium powders to nanoparticle sizes. Also, the crystal sizes and the changes in the microstructures of the ethanol wet-milled niobium were determined. However, it is noteworthy to state the fact that the authors could not find any research work on the use of ethanol to mill pure niobium flakes or powders, hence, this study. Besides, the purpose of doing this research is to increase the surface area of the niobium particles for better performance. A nano-sized particle of niobium has a greater surface area than the same niobium as a micro-size particle. To increase the surface area of the niobium particles is to increase the number of catalytic sites to enhance good adhesions of the particles during reactions.

## Experimental

**Figure 1:**
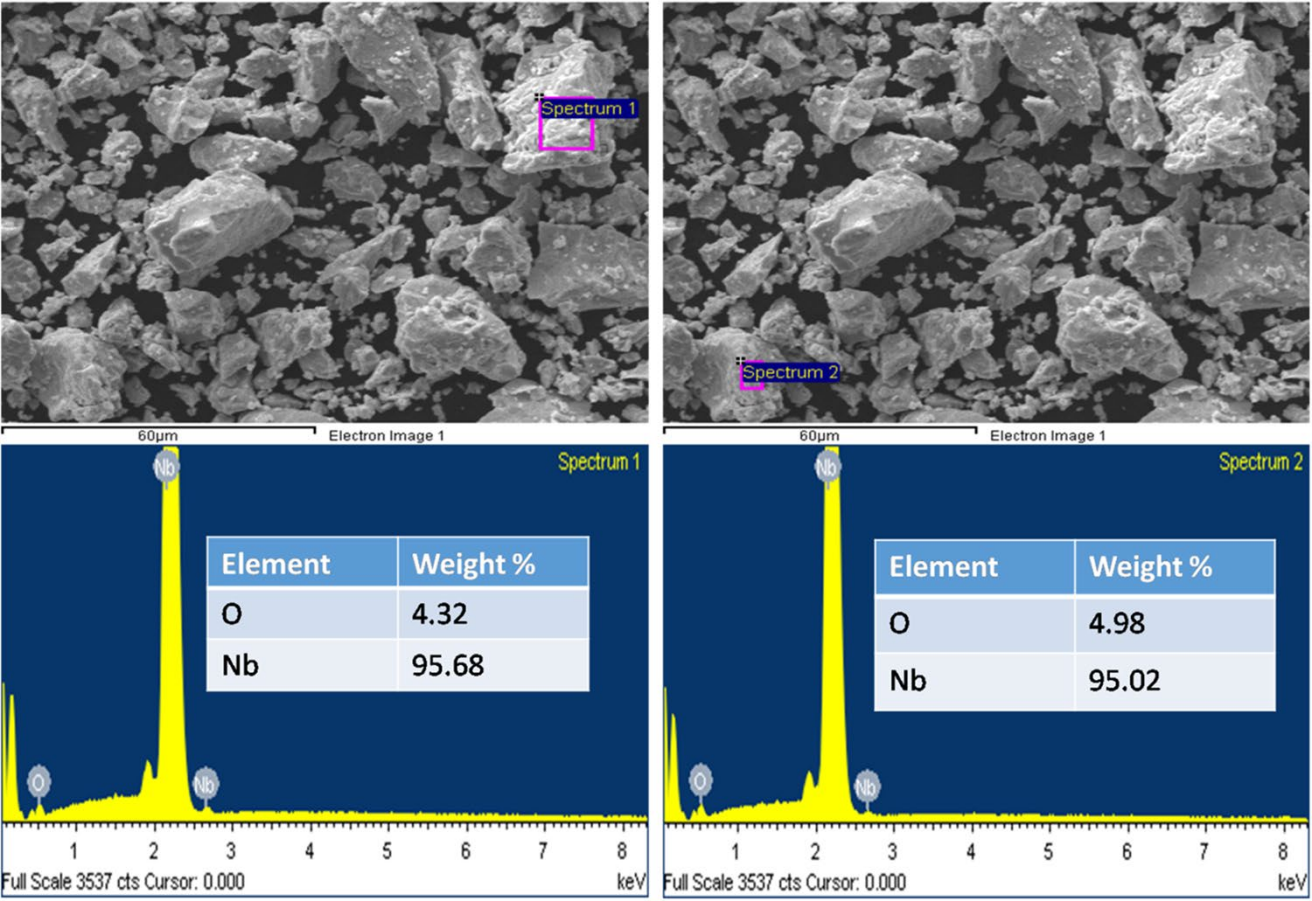
shows the SEM and EDS images of the starting powders.

In the current study, the raw material used is pure commercial Nb powder (95.7% pure) with a particle size of ~ 44 µm that was supplied by Alfa Aesar Company. Milling was performed under the condition of a wet ball milling process. For the milling process, 40.12 g of the as-received Nb powder were loaded into two separate hardened steel containers of 125 ml volume with steel balls of 0.4 mm diameter, in a ball-to-powder weight ratio of ~ 10:1.80% volume of the hardened steel containers were filled with 5 ml of absolute ethanol that served as the process-controlling agent. The containers were closed and weighed, to balance their weights and to avoid imbalance in the milling machine. The powder was wet-milled for 10 h at a speed of 400 rpm, at an interval of 15 min, and with an interval break of 5 s. After the wet milling procedure, the powders were recovered through drying in a vacuum oven, at a temperature of 100 °C, a speed of 15 rpm, a time of 653 min, and in vacuum at 250 mbar. The crystal phase of the dried powders was analyzed by using the X-ray diffraction (XRD) analyzer, the EMPYREAN DIFFRACTOMER with CuK_ɑ_ radiation and was analyzed by using the Highscore with software. The morphology and the elemental compositions of the dried powders were investigated by using the High-Resolution Field Emission Scanning Electron Microscope (SEM), (JEOL-JSM-7600F), attached with energy dispersive x-ray spectroscopy (EDS) (Fig. 1).

## Results and discussion

### XRD results

**Figure 2:**
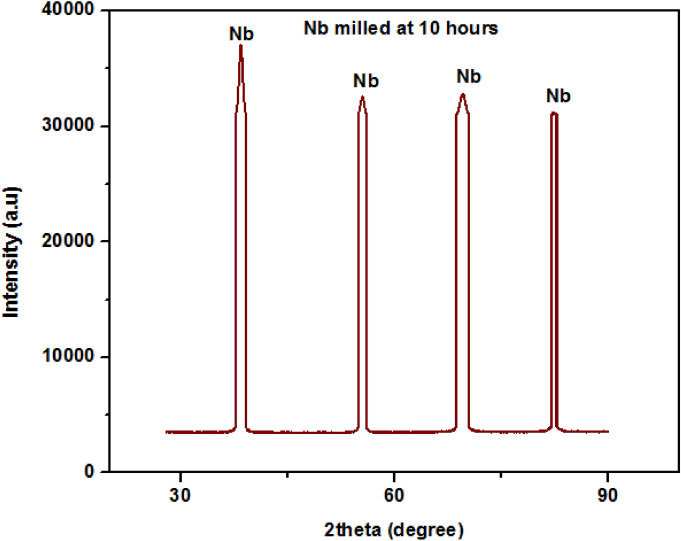
XRD diffraction pattern of the milled Nb powder.

**Figure 3:**
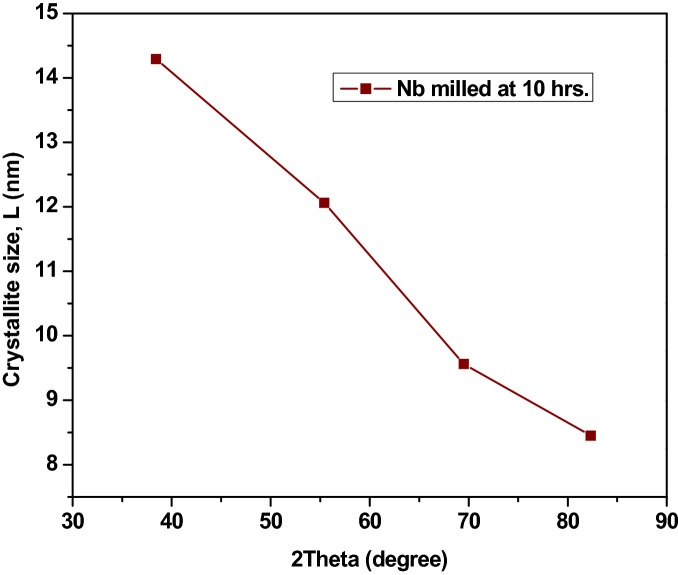
The plot of Crystallites size, L as a function of 2θ.

**Figure 4:**
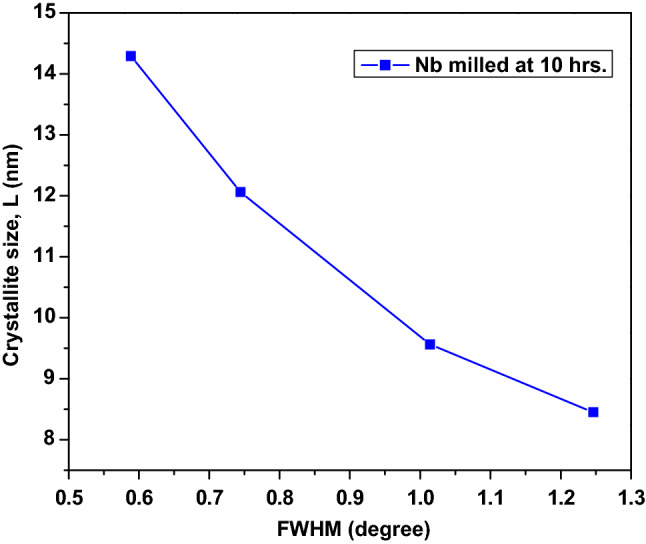
Plot of the crystallite size, L as a function of the FWHM of the peak profile.

The XRD diffractograph of the milled Nb powder is shown in Fig. 2, consisting of four sharp peaks. The sharpness of the peaks decreased with an increase in 2θ, which indicated a decrease in the crystal size of the powders. Table 1 summarises the Scherrer equation data for the milled powders. In the Table, there is a moderately gradual increase in the values of β · cosθ and 2θ with decreases in the crystal size, *L* values. Figure 3 shows the graph of the crystal size of the milled powders against the angle between the transmitted beam and the reflected beam, 2θ and Fig. 4 shows the graph of the crystal size, *L*as a function of FWHM of the peak profile. It was observed that there is a decrease in the crystal sizes as the 2θ and FWHM values increase; this behavior might be a result of the crystalline nature of the Nb powdered material.

**Table 1:** The summary of the Scherrer equation data for the milled powders.

Peak Position [°2θ]	FWHM [°]	β = FWHM [in radians]	Bragg angle θ [°]	Cosθ	β · Cosθ	Crystallite size, L [nm]
38.4231	0.5884	0.0103	19.2116	0.9443	0.0097	14.2900
55.4389	0.7446	0.0130	27.7195	0.8852	0.0115	12.0600
69.5056	1.0138	0.0177	34.7528	0.8216	0.0145	9.5600
82.3355	1.2465	0.0218	41.1678	0.7528	0.0164	8.4500

**Figure 5:**
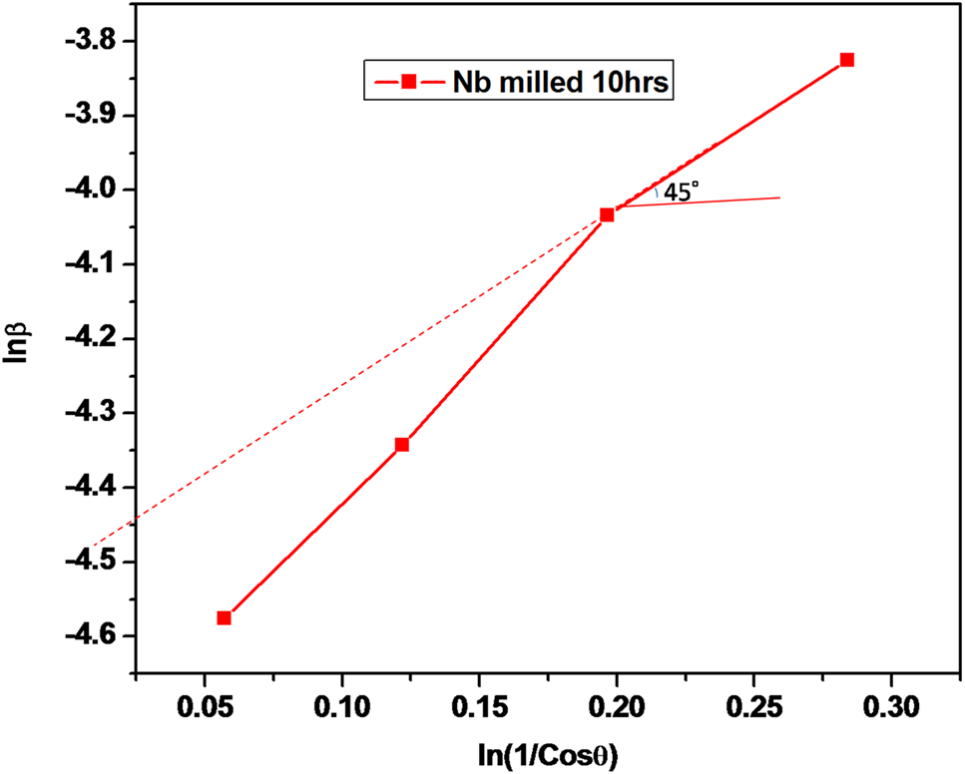
Modified Scherrer’s equation (the graph of lnβ against ℓn(1/cosθ)).

Table 2 is the summary of the modified Scherrer’s equation data (values of ℓnβ and ℓn(1/cosθ)) and Fig. 5 shows the modified Scherrer’s equation (the plot of ℓnβ as a function of ℓn(1/cosθ)) of the milled powders. Since β · Cosθ value is, in fact, not a constant value for all the four peaks, this was the cause of the deviation from a 45° slope, observed in Fig. 5. The slope of the modified Scherrer’s equation plot in this study is negative. This is as a result of the fact that at high 2θ angles, with low values of Cosθ and higher values of ℓn(1/Cosθ), the sizes of the β values obtained are less than what it must be when applied to the Scherrer’s Eq. ^20^. The modified Scherrer equation can offer the advantage of reducing the size of the absolute values, thereby, producing a single line throughout the points and hence, yielding a single value of the intercept, where a single value of the crystal size, *L*, of the available peaks, can be obtained^20^.

**Table 2:** The summary of the data obtained from the modified Scherrer equation (values of lnβ and ℓn(1/cosθ))of the milled powders.

β (radian)	ℓnβ	Cosθ	1/Cosθ	ℓn(1/Cosθ)
0.0103	− 4.5756	0.9443	1.0590	0.0573
0.0130	− 4.3428	0.8852	1.1297	0.1220
0.0177	− 4.0342	0.8216	1.2171	0.1965
0.0218	− 3.8258	0.7528	1.3284	0.2840

Monshi et al.^20^, reported the fact that if the result of ℓnβ is plotted as a function of ℓn(1/cosθ), a straight-line graph with a slope of around one and an intercept of about ℓn $\frac{K}{L}$, should result. After obtaining the intercept, the exponential of the intercept is obtained, which is equal to $\frac{K\lambda }{L}$ and knowing the values of *K* and ʎ, a single value of *L* in nanometer, can be calculated^20,22^. However, in this study, Fig. 5 is the plot of ℓnβ as a function of ℓn(1/cosθ). The intercept of the resulting graph is:

$${4}.{4469}\;{\text{and}}\;\frac{K\lambda }{L} = {\text{ e}}^{{ - {4}.{4469}}} .$$

Therefore, $$\frac{K\lambda }{L} = 0.0{117}.$$

The single crystallite size, L of the four peaks is,

$${\text{L}} = \frac{{\left( {0.9} \right)\left( {0.15405} \right)}}{0.0117}$$

Therefore, the single average crystallite size of the whole peaks is,

$${\text{L}} = {11}.{85}\;{\text{nm}}.$$

### SEM and EDS of the milled Nb powder

**Figure 6:**
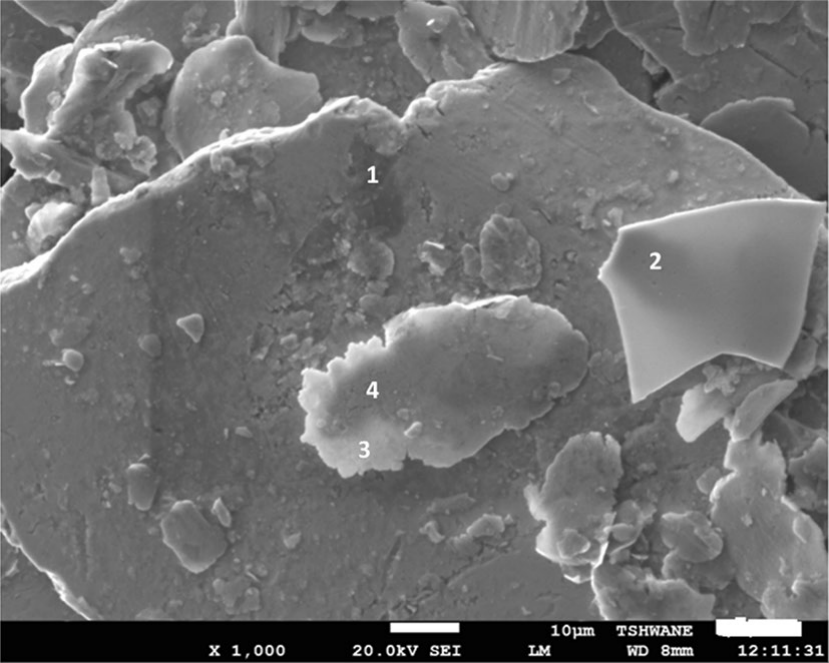
SEM of the milled Nb powder.

**Figure 7:**
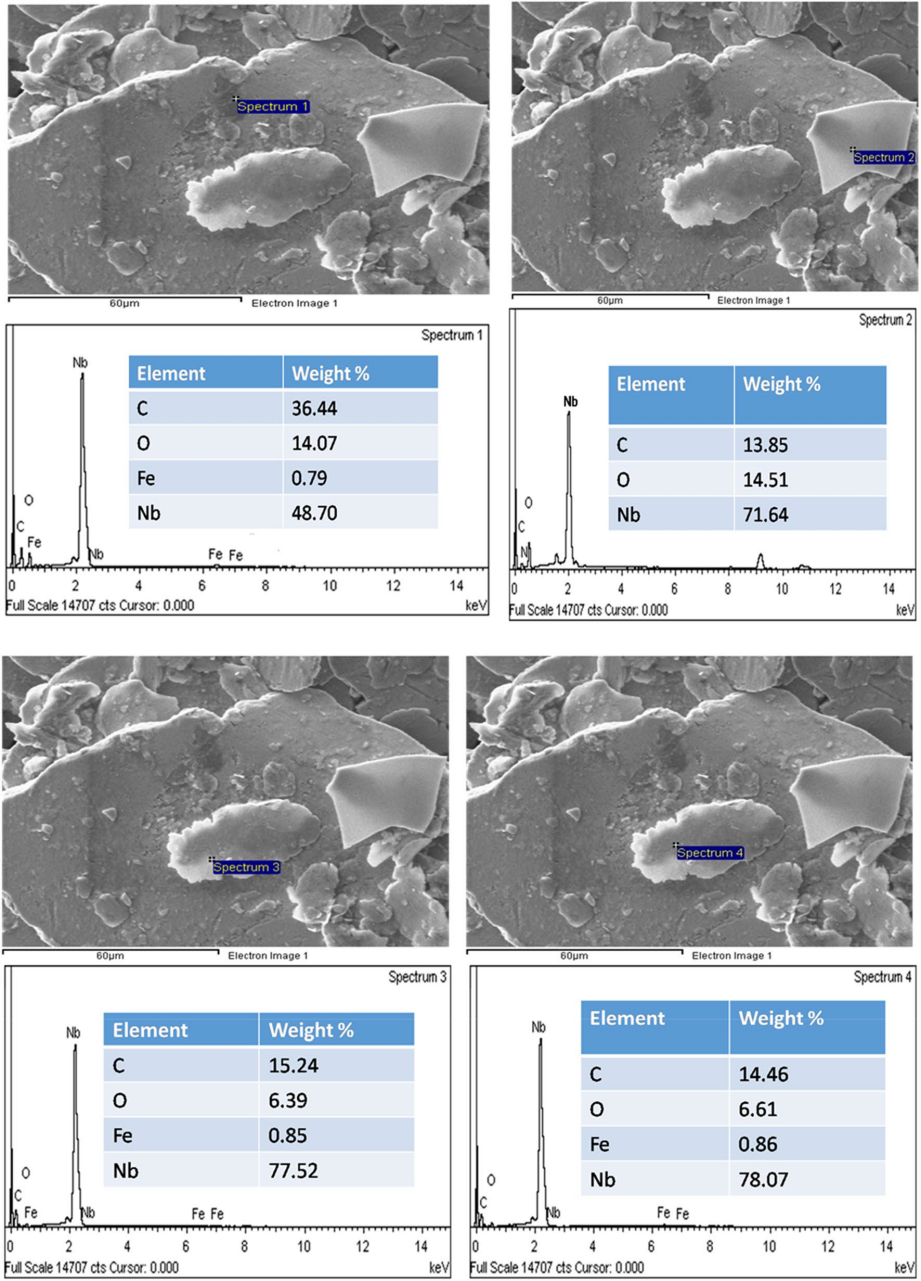
EDS element composition of the milled Nb powder.

Figure 6 shows the SEM of the milled Nb powder. The points labeled: 1, 2, 3 and4are the spectra identified by the EDS (Fig. 7). The microstructure image in Fig. 6 shows that ethanol wet milling can reduce the particle sizes and minimize the rate of agglomeration of the Nb powders after milling. The particle size of the powder, slowly decreased with the increasing rotation speed, from 200 to 400 rpm of the milling machine, owing to the high energy milling process^23^. During the ethanol wet milling process, the Nb powder, ethanol, and the steel balls were subjected to high energy particle-to-particle interaction, and the steel milling balls collision at a high revolution speed of 400 rpm. It appeared achievable that these outcomes are appropriate to the initial grains, which were broken down into unit squashed portions. However, looking at the upper left side of the SEM image (Fig. 6) it can be observed that the powder attained a certain level of agglomeration after 10 h of the ethanol-wet milling. Hence, more time is required in achieving, often tiny particle sizes, during the ethanol wet milling of the Nb powder. The high definite surface area of the particles increases the van der Waals forces between them and this is the basis of the de-agglomeration of the powdered particles^6,24^, following wet milling.

This present work is significant, considering the possibility of reducing the particle size and the minimization of agglomeration, which will result in the better sintering of the Nb powders. Also, Nb being a good alloying element, the enhancement of the particle surface area will influence the interfacial-adhesion of the Nb powder particles, which enable them to marry well with the particles of the base element (different material). The results of the current study are consistent with those of Fayyaz et al.^6^, Yang and German^25^, who found out that wet milling is appropriate to decrease the rate of agglomeration and reduce the particle size of pre-alloy nanophase metallic powders. Figure 7 shows the EDS elemental compositions of the ethanol wet-milled Nb powder. Fe, O, and C were observed as impurities in the EDS analysis. The Fe presence may be from the steel balls used in the milling since Fe is the major component of steel. The presence of oxygen could be due to the native oxide layer at the surface of Nb (Fig. 1), which then enhances the admission of more oxygen from the processing liquid (ethanol). It is frequently mentioned in the literature, in the study of niobium that the existence of a native oxide layer on the surface of Nb, exposed it to oxygen, and Nb is characterized by its high attraction and binding energy to oxygen^26–28^. To obtain a highly pure Nb surface, without the presence of oxygen or oxide layer, the metal powder was annealed or heated above the temperature of 2000 K, in ultra-high vacuum conditions^11,26,27^. A significant presence of carbon, C (up to 36 wt.%) was observed in the milled powder when compared to other impurities (Fe = 0.8 wt.%, O = 14 wt.%) and this is purely from the ethanol used in the milling. Nb can form very stable carbides, possessing high inter-atomic bonding energy and it is used in the stabilization of stainless steels to prevent intergranular corrosion since it helps to lower the content of carbon in the steel^27,29^. The relevance of this study cannot be overemphasized, since the results of this study, have shown another means of extracting important and useful transition carbide (niobium carbide, NbC).

## Conclusion

1. The use of ethanol in the wet milling of Nb powder enhanced the reduction of the particle sizes, from -44 µm to an average crystallite size of 11.85 nm, according to the modified Scherrer’s equation employed in the determination of crystal size. The application of the Scherrer’s equation systematically shows increases in the values of nano crystallite size as FWHM and 2θ values decrease since β · cosθ cannot be maintained as constant.
2. In the plot of ℓnβ as a function of ℓn(1/cosθ), the exponent of the intercept was equal to $\frac{K\lambda }{L}$ , from which a single average value of L = 11.85 nm was obtained.
3. The study can be another way of synthesizing important transition carbide, e.g., NbC, as exemplified in the SEM and EDS results.
